# Mental health impacts of climate change on vulnerable populations in Nigeria and Japan: A systematic review of access to emergency healthcare

**DOI:** 10.1016/j.puhip.2026.100804

**Published:** 2026-05-08

**Authors:** Adewunmi Oluwaseun Adebayo, Ramita Thawonmas, Miho Sato

**Affiliations:** a Interfaculty Initiative in Planetary Health (IIPH), Nagasaki University, Nagasaki, Japan; b School of Tropical Medicine and Global Health (TMGH), Nagasaki University, Nagasaki, Japan

## Abstract

**Objective:**

This systematic review synthesises evidence on the mental health impacts of climate change on vulnerable populations in Nigeria and Japan and evaluates their access to emergency healthcare services.

**Study design:**

Systematic review of peer-reviewed and grey literature.

**Methods:**

Following PRISMA 2020 guidelines, we searched MEDLINE, PsycINFO, PubMed, CINAHL, and grey literature (2015 to March 2025) for studies on climate change, mental health, and healthcare access. Inclusion criteria encompassed primary studies of vulnerable populations (e.g., displaced persons, rural women, elderly) exposed to climate-related events. Two reviewers independently screened titles/abstracts and full texts, appraised quality using CASP checklists, and extracted data. A narrative synthesis was conducted due to methodological heterogeneity. PROSPERO registration: CRD420250651981.

**Results:**

Of 143 records identified, 8 studies (Nigeria: 5 qualitative; Japan: 3 quantitative) met inclusion criteria. In Nigeria, climate-induced displacement, flooding, and livelihood loss exacerbated anxiety, depression, and PTSD, compounded by gender disparities and inadequate healthcare infrastructure. In Japan, heatwaves increased heatstroke incidence among elderly populations, with mental health risks linked to social isolation and delayed care-seeking. Both settings highlighted systemic barriers: Nigeria's underfunded mental health services and Japan's stigma-related underutilization of care. CASP appraisal rated studies as moderate-to-high quality.

**Conclusions:**

Climate change disproportionately affects mental health in vulnerable populations, with context-specific drivers in low-versus high-income settings. Policymakers must integrate mental health into climate adaptation strategies, prioritising gender-sensitive interventions in Nigeria and age-targeted emergency responses in Japan. Strengthening healthcare access and addressing socio-cultural barriers are critical to mitigating climate-related psychological burdens.

## 1 Introduction

Global warming is one of the major world issues and concerns everyone since it influences the environment and health. This implies that cardiovascular and mental diseases can be aggravated by climate conditions. Increased sea levels and displacements, which force people to leave their homes, also contribute to the increase in mental health problems around the world. Although climate change has natural components, current changes are primarily anthropogenic, and largely driven by human activities such as fossil combustion, gas emissions, and deforestation [1]. Africa and South Asia are most affected by climate change as their agricultural and fishery production is being reduced [2]. People of low income and those who have anxiety disorders are at a greater risk of climate change-influenced diseases [3]. Apart from day-to-day disasters such as floods and heat waves, climate change results in food and water deprivation and forced migration, which in turn are associated with increased prevalence of mental health disorders such as anxiety and depression [4,5].

Socioeconomic status and education also raise the likelihood of developing health complications and escalate the risk since they cause vulnerability to complex diseases [6,7]. In Nigeria, flooding and extreme temperatures of a daily average of 26 °C and above and below 18 °C in rural areas result in respiratory and cardiovascular diseases, famine, and depression [8,9]. Likewise, in Japan, even the most developed healthcare systems suffer from the effects of natural calamities like earthquakes that worsen anxiety and depression if not adequately addressed [10]. These events harm livelihood, lead to financial pressure, and limit access to mental health services [11]. The loss and displacement of people's homes due to disasters in Japan threaten their lives, and their mental health suffers for years because of the possible recurrence [12]. However, the responses of the healthcare systems are different. In Nigeria, particularly, the funding is low, and very few health professionals in psychiatry are available; people with mental health issues receive little or no support at all [13]. In Japan, modern amenities are provided, but the prejudice against mental illness compels people not to seek treatment [10].

Mental health among vulnerable population groups is easily affected by climate change; this includes participants who are suffering from depression and anxiety. The studies indicate how climate change is detrimental to the mental health of a populace, but the research on how those with such disorders are affected is inadequate [14]. Factors, including stigma, cost, and availability of services, influence mental health as far as health determinants are concerned [15]. This is due to factors that include age, income, geographical location, and a shortage of healthcare facilities in Nigeria [16]. Likewise, traditional values regarding patient confidentiality and internal sensitisation in Japan hinder appropriate healthcare [17]. This review aims to systematically evaluate evidence on the impact of climate change (Exposure) on the mental health (Outcome) of vulnerable populations (Population) and their access to emergency healthcare services in Nigeria and Japan.

This research aims to fill the knowledge gaps concerning climate change's effects on mental health and healthcare access among vulnerable groups. Using Nigeria and Japan as examples, the study illustrates the problems developing and developed nations face. The report will offer practical guidelines that will serve as a reference for policymakers and healthcare agencies to design focused prevention strategies, understand the factors influencing poor mental health, address them, and enhance equal access to emergency care services. Additionally, analysing the results will be beneficial for taking action in other countries with similar problems. The research will provide a comparative analysis of the effects of climate change on the mental health of vulnerable groups in Nigeria and Japan. However, it does not extend to other aspects of health and well-being, such as physical and social well-being. The findings are therefore valuable for countries facing similar problems, though not generalisable due to cultural differences, economic status, and geographical location.

## 2 Methods

### 2.1 Study design

We used the reporting items for systematic reviews and meta-analysis (PRISMA) checklist for 2020 for this systematic review [18]. Thus, the PRISMA checklist is essential for explaining the approach used to identify themes and gaps in the literature. The PRISMA checklist also contributes to the validity of several evidence [[19], [20]]. However, the PRISMA guideline provides a framework that enhances data transparency. This systematic review was registered in PROSPERO (Registration ID: CRD420250651981), ensuring transparency and methodological rigour from the outset. It is thus essential to note that the PRISMA checklist improves the process of identifying and collecting articles.

### 2.2 Data sources and search strategy

A systematic search was conducted in four electronic databases: Web of Science, Medline, PsycINFO, and CINAHL, to identify original studies published between January 2015 and March 2025. This timeframe, in line with PRISMA recommendations [18], was chosen to ensure the inclusion of recent and relevant research while maintaining repeatability.

The search focused on studies examining the mental health of vulnerable populations in the context of climate change and emergency response. Priority was given to peer-reviewed articles published in English to minimize the risk of omitting high-impact studies, as many influential articles are published or translated into English. The search strategy combined relevant keywords and controlled vocabulary (subject headings), including terms such as:

(“Displaced populations” OR “Climate refugees” OR “Internally displaced people” OR “Climate-induced migration” OR “Forced migration”) AND (“Emergency healthcare” OR “Access to healthcare” OR “Healthcare services” OR “Medical services” OR “Healthcare accessibility” OR “Proximity to healthcare” OR “Affordability of healthcare” OR “Quality of healthcare”) AND (“Adequate healthcare access” OR “Improved access to healthcare” OR “High-quality healthcare services” OR “Affordable and available healthcare”) AND (“Mental health” OR “Psychological health” OR Anxiety OR PTSD OR Depression OR Stress OR “Mental health outcomes”)

To enhance the thoroughness of the search, hand-searching of reference lists from retrieved articles and consultation with subject matter experts were conducted. By applying these search parameters and adhering to PRISMA guidelines [18], the review aimed to compile a robust body of evidence on the intersection of climate change, mental health, and emergency healthcare access among vulnerable populations.

### 2.3 Study inclusion and exclusion criteria

This review applied specific inclusion and exclusion criteria to identify relevant quantitative research on the mental health impact of climate change on vulnerable populations and their access to emergency healthcare services (Appendix 1). Only studies published in English were included. Exclusion criteria encompassed studies that did not focus on the mental health effects of climate change or emergency healthcare access, articles with fewer than two study subjects, review articles, unfinished studies, conference abstracts, and editorials. The PEO framework was utilised to structure the research questions and guide the selection of relevant studies [19]. This framework helped define the target population (vulnerable populations), the exposure (climate change), and the outcomes (mental health impact and access to emergency healthcare services) [19]. The review question and objectives were aligned with the PEO framework, and only studies meeting the following criteria were included:

• **Population (P):** Studies focusing on vulnerable populations affected by climate change, presenting primary data with more than two study subjects.
• **Exposure (E):** Factors or characteristics associated with climate change that influence mental health outcomes and access to emergency healthcare services.
• **Outcome (O):** Studies examining the relationship between climate change, mental health impacts, and access to emergency healthcare.

### 2.4 Selection and appraisal

#### 2.4.1 Study selection

The selection process followed a structured approach based on the PRISMA framework, consisting of four key stages: identification, screening, eligibility, and inclusion. The search results from the selected databases were exported to EndNote to facilitate efficient organisation and the removal of duplicate articles. Duplicates were carefully reviewed and removed to ensure each study was counted only once during the selection process. The remaining unique articles were transferred to Microsoft Excel for further screening, leveraging the platform's filtering and sorting capabilities to aid selection. Two reviewers independently screened titles, abstracts, and full texts against the established inclusion and exclusion criteria. Disagreements were resolved through discussion and, where needed, by a third reviewer to ensure consistency and minimize bias. Subsequently, eligible studies then underwent a full-text review to confirm inclusion.

#### 2.4.2 Data extraction

The data extraction process is a vital step in a systematic review, facilitating the presentation and synthesis of information from the included studies [21]. This process ensures that all findings from the selected studies are systematically gathered and synthesised following a quality assessment [22]. Data extraction was conducted using a pre-tested data extraction form based on the PEO framework, capturing key details such as study design, study population characteristics, and significant findings across the included studies. Two reviewers independently extracted data, with a random sample of articles cross-verified to ensure consistency. The synthesised information from the selected studies is presented in the data extraction table.

#### 2.4.3 Quality assessment

This systematic review employed the Critical Appraisal Skills Programme (CASP) checklist, a comprehensive framework for evaluating research quality, using the CASP Checklist for Qualitative Studies to assess the quality of the qualitative studies (Appendix 3) and the CASP Checklist for Cohort Studies to evaluate the quality of the quantitative studies (Appendix 4). Two reviewers independently performed the quality assessment, and discrepancies were resolved through discussion and re-appraisal to ensure reliability.

Qualitative studies scored 7-10 (moderate to high quality). The assessment revealed that most qualitative studies provided clear research aims, appropriate methodologies, and well-justified data collection techniques. However, some studies lacked sufficient details on researcher reflexivity and potential bias. Quantitative studies scored 9-12, indicating high methodological rigour. The majority of these studies demonstrated robust research designs, appropriate statistical analyses, and clearly defined research questions. However, limitations were noted in controlling for confounding variables and in the generalizability of findings, particularly in studies with smaller sample sizes or non-representative populations.

Overall, the CASP quality assessment indicates that both the qualitative and quantitative studies included in this review were of high quality, thereby strengthening the reliability of the evidence synthesised. Additionally, Tran et al. noted that quality assessment is crucial for determining the overall strength of studies, minimising bias, and ensuring a rigorous and objective evaluation of research methods and findings [23].

## 3 Results

### 3.1 Study selection

**Fig. 1:**
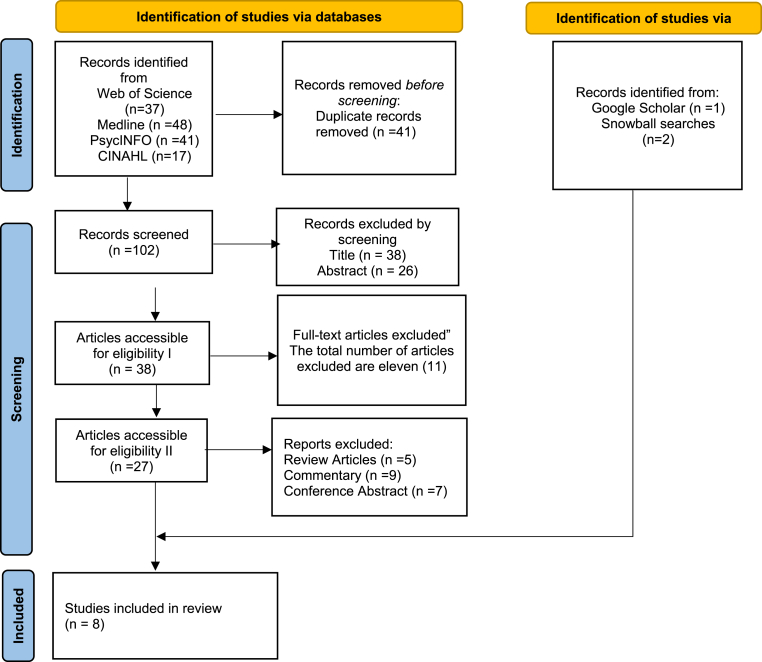
PRISMA flowchart.

The initial search identified a total of 143 records across four major databases (Web of Science, PsycINFO, MEDLINE, CINAHL) and relevant grey literature sources. After removing 41 duplicates, 102 records were screened by title and abstract. As a result, 64 studies were excluded. Full-text screening was conducted for 38 studies, of which 11 were excluded for not meeting the inclusion criteria. This resulted in the inclusion of five studies from the database search. Additional records were identified through snowball searching, Google Scholar, and reference list reviews, yielding three more studies. In total, eight studies were included in this systematic review. The PRISMA flowchart (Fig. 1) summarises the selection process. The search was restricted to studies published within the past 10 years (January 2015 – March 2025), with no language restrictions beyond English and Japanese. Experts in climate-related displacement and emergency healthcare were consulted to identify unpublished research.

### 3.2 Study characteristics

This systematic review included eight studies (Appendix 2), conducted between 2015 and 2024, examining the impact of climate-related health risks and extreme weather events on vulnerable populations in both Nigeria and Japan. The populations studied included internally displaced persons (IDPs), rural women, riverine traders, and heatstroke-affected individuals.

The methodological approaches comprised qualitative studies (n = 4), phenomenological studies (n = 1), case-crossover studies (n = 2), and time-series studies (n = 1). The studies addressed climate change-related health challenges, such as vulnerability to disease, livelihood disruptions, and heat-related illnesses. Sample sizes ranged from 32 participants [24] to 12,907 cases [25].

Studies in Nigeria emphasised flooding, displacement, and gender-related vulnerabilities, while those in Japan focused on heatstroke incidence and environmental determinants with urban and ageing contexts. Due to methodological heterogeneity, a meta-analysis was not feasible; instead, a narrative synthesis was employed to integrate findings across diverse study designs and settings thematically. This approach was particularly appropriate for exploring qualitative and observational outcomes that could not be statistically pooled.

Variations in study populations and settings, ranging from internally displaced women in Nigeria to elderly urban residents in Japan, were considered during synthesis and interpretation. These differences in demographic, cultural, and environmental contexts may influence the generalizability of findings and highlight the importance of localised adaptation strategies. The characteristics and outcomes of the eight included studies are summarised in Appendix 2.

### 3.3 Impact of climate change on mental health among vulnerable populations

The studies reviewed highlight that climate change is a significant driver of mental health outcomes among vulnerable populations in Nigeria and Japan. In Nigeria, evidence from internally displaced persons (IDPs) in Lagos [24] and rural women in Anambra State [26] revealed that climate-related events, especially flooding and displacement, are linked with psychological distress. Thus, it is vital to note that these climate-induced disruptions contribute to an increased level of anxiety, depression and post-traumatic stress, which is largely attributed to loss of livelihoods, housing instability and limited access to healthcare services. These findings, along with others detailed in Appendix 2, show how climate stressors translate into measurable mental health burdens in diverse at-risk populations. In Japan, the association between climate change and mental health is observed using heat-related exposures. Studies on heatstroke incidence [[27], [28], [29]] provide evidence that rising temperatures and consistent heatwaves contribute not only to physical health risks but also to psychological strain. Also, among older adults in Tottori and Fukuoka, continuous exposure to extreme heat and urban heat effect is linked to increased anxiety, stress and cognitive decline [29]. Therefore, suggesting that climate-induced environmental conditions, especially heat stress, are an indirect determinant of mental health outcomes.

### 3.4 Access to emergency healthcare services in climate context

Climate-induced disruptions to healthcare access were observed across both countries, with significant disparities in emergency response capacity. In Nigeria, women traders in flood-prone riverine areas [30] reported delays in receiving medical care due to infrastructural damage and economic constraints. Similarly, Duru et al. found that rural women in Kwara State experienced barriers to emergency services due to inadequate transportation networks and resource limitations [30]. Conversely, in Japan, advanced emergency response systems have mitigated some impacts of climate-related health crises. Studies on heatstroke response in Tottori and Fukuoka [27,29] highlight the role of meteorological data and pre-emptive healthcare measures in reducing severe outcomes.

### 3.5 Socioeconomic and environmental determinants of climate-related mental health

#### 3.5.1 Outcomes

Across both Nigeria and Japan, multiple socio-environmental factors modulate the mental health impact of climate change. In Nigeria, gender disparities and economic instability emerged as key determinants. Akanwa et al. found that women faced compounded vulnerabilities due to caregiving responsibilities, limited financial autonomy, and exposure to climate-related violence [26]. Similarly, Micheal reported that female traders struggled with increased caregiving burdens and economic precarity, further exacerbating mental health outcomes [30]. In Japan, age and urbanisation levels were significant moderators of heat-related health risks. Toosty et al. identified individuals aged 70 and above as particularly vulnerable to severe heatstroke, with increased hospitalisation rates linked to prolonged exposure to high wet-bulb globe temperatures [29].

#### 3.5.2 Comparative analysis and policy implications

The comparative evidence shows that although the pathways are different climate change affects mental health in both Nigeria and Japan using context specific mechanisms. In Nigeria, the mental health burden is driven by displacement, poverty and weak healthcare systems, while in Japan it is linked to environmental stressors like extreme heat and demographic vulnerabilities. These findings highlight the need for targeted, policy-driven interventions. In Nigeria, it is important to strengthen healthcare infrastructure using mobile health clinics and community-based mental health programmes to improve access during climate emergencies [31]. In Japan, integrating mental health support into heat emergency response frameworks will help address the psychological dimensions of climate exposure, especially among older adults [32]. Overall, the evidence illustrates that climate change is not only an environmental and physical health problem but a major determinant of mental health [33]. Thus, addressing its impacts necessitates a multisectoral approach that combines climate adaptation strategies with accessible and equitable mental health services.

## 4 Discussion

The findings from this review highlight the significant impact of climate change on mental health in Nigeria and Japan, emphasising the interplay between environmental stressors and psychological well-being. In Nigeria, climate-related mental health outcomes are mainly driven by acute environmental disruption like flooding, displacement, and economic instability. These stressors contribute to elevated levels of anxiety, depression, and post-traumatic stress disorder (PTSD) among vulnerable populations, particularly women and internally displaced persons [26]. In contrast, in Japan, the mental health burden is more closely associated with chronic environmental exposure, particularly prolonged heatwaves and urban heat island effects, which disproportionately affect older adults and are linked to anxiety, stress, and cognitive decline [29]. This distinction highlights a key comparative insight: while climate change in Nigeria manifests through sudden, high-impact events that destabilise livelihoods, in Japan it operates through gradual but persistent environmental stressors that accumulate over time. These differing exposure pathways shape not only the type but also the severity and duration of mental health outcomes.

Comparison with studies from other regions reinforces both shared and context-specific dynamics. In high-income countries, extreme events such as wildfires have been associated with increased PTSD and substance use disorders, particularly in previously exposed populations [34]. Meanwhile, evidence from African contexts indicates that food insecurity and water scarcity are critical mediators of climate-related psychological distress [35]. This suggests that resource availability and baseline socioeconomic conditions play a central role in determining how climate stress translates into mental health outcomes.

Another important finding is the limited use of clinically validated mental health measures across the reviewed studies. Most studies rely on self-reported psychological distress or proxy indicators rather than formally diagnosed psychiatric conditions. This lack of standardised measurement makes it difficult to directly compare the magnitude of mental health impacts between Nigeria and Japan and contributes to the reviewer's concern about measurement. It also highlights a broader gap in the literature, particularly in non-Western settings, where diagnostic data on climate-related mental illness remains scarce.

A further point of divergence lies in healthcare system capacity and access. Nigeria faces systemic infrastructural and financial constraints that limit emergency healthcare delivery during climate crises, thereby exacerbating both physical and psychological vulnerability. In contrast, Japan's advanced emergency response systems mitigate immediate health risks; however, gaps remain in addressing the longer-term mental health consequences, particularly among socially isolated elderly populations. This suggests that while health system strength influences outcomes, it does not fully eliminate vulnerability, as behavioural and social factors also play critical roles.

Socioeconomic determinants further mediate these impacts in distinct ways [36]. In Nigeria, gender inequality amplifies mental health risks, with women experiencing compounded stress due to caregiving responsibilities, financial insecurity, and exposure to climate-related violence [26]. In Japan, demographic ageing is the dominant vulnerability factor, with older adults facing increased susceptibility to heat-related stress due to physiological limitations and social isolation [29]. Similar patterns have been observed in South Asia, where women and older adults disproportionately bear the mental health burden of climate change due to entrenched social inequalities [37]. From a comparative perspective, these findings underscore that climate change does not produce uniform mental health outcomes; rather, its effects are mediated by context-specific social, economic, and infrastructural conditions.

From a policy standpoint, there is an urgent need to integrate mental health into climate adaptation strategies in both settings. However, interventions must be tailored: in Nigeria, strengthening healthcare infrastructure and implementing gender-sensitive, community-based mental health programmes are critical, whereas in Japan, policies should prioritise age-responsive interventions and the integration of mental health services into heatwave preparedness frameworks.

## Ethical statement

Ethical approval was not required for this study, as it is a systematic review of previously published literature and does not involve any primary data collection or human participant recruitment. All data analysed were obtained from publicly available studies.

## Funding statement

This work was supported by the Nagasaki University WISE Programme.

## Competing interests

The authors delcate that they have no competing interests.

## Figures and Tables

**Table:** 

PEO Elements	Inclusion Criteria	Exclusion Criteria	Justification
Population	Vulnerable populations affected by climate change, presenting primary quantitative data with more than two study subjects.	Studies not focusing on vulnerable populations, case reports with fewer than two participants, and review articles.	Ensures that the review captures empirical evidence directly addressing the experiences of vulnerable populations, avoiding anecdotal or secondary findings.
Exposure	Any factor or characteristic related to climate change that influences mental health outcomes and access to emergency healthcare services.	Studies that do not address climate change as a determinant, or focus only on unrelated health or environmental factors.	Maintains focus on climate change as the key exposure, ensuring relevance to the review's objectives.
Outcomes	Quantitative evidence on the relationship between climate change, mental health impacts, and access to emergency healthcare.	Studies that exclude mental health or emergency healthcare access, or examine only physical health outcomes without mental health considerations.	Guarantees that both mental health impacts and healthcare access are examined, aligning with the review question.
Other Criteria	Peer-reviewed quantitative studies published in English, reporting complete and finalised findings.	Studies in other languages, unfinished studies, conference abstracts, editorials, and commentaries.	Ensures methodological rigour and accessibility, while excluding incomplete or non-peer-reviewed sources.

**Table:** 

Author (Year)	Location	Study Population	Sample Size	Type of Study	Methods	Outcome Variable	Moderator Analysis	Symptom or diagnosis Type	Exposure Type	Timeframe of Outcomes Evaluated
Adejoh et al. (2022) [24]	Lagos, Nigeria	Internally Displaced Persons (IDPs)	32	Qualitative Study	Interviews, Focus Group Discussions	Vulnerability to COVID-19, Healthcare Access	N/A	Health Conditions (Sanitation, Overcrowding, Access to Care)	COVID-19 Pandemic, Lockdown Effects	Early Stages of the COVID-19 Pandemic (2020)
Akanwa et al. (2022) [26]	Anambra State, Nigeria	Rural Indigenous Women	N/A	Qualitative Case Study	Field Observation, Transect Walks, Photography, Random In-depth Interviews, Focus Group Discussions	Impact of long-term flooding on health, livelihoods, and gender inequalities	Gender, Cultural Norms, Resource Distribution	Health Conditions (Injuries, Malaria, Waterborne Infections, Sexual Harassment)	Health Conditions (Injuries, Malaria, Waterborne Infections, Sexual Harassment)	Long-term Flooding, Climate Change
Duru et al. (2022) [38]	Ilorin South, Kwara State, Nigeria	Rural Women	270	Phenomenological Study	Questionnaires, Interviews, Focus Group Discussions	Impact of Climate Change on Livelihood	Lack of Basic Infrastructure, Low Capital, Crude Farming Methods	Livelihood Disruption, Resilience, Vulnerability to Climate Change	Climate Change Disasters	Ongoing Impact
Micheal (2024) [30]	Bayelsa State, Nigeria	Women Traders in Riverine Areas	46	Qualitative Study	In-depth Interviews [39], Focus Group Discussions [3], Thematic Analysis	Impact of Climate Change on Women Traders' Livelihoods	Gender, Economic Disparities, Social Inequalities	Livelihood Disruptions, Market Access Barriers, Increased Caregiving Burden	Climate-related Flooding	Ongoing Impact
Otani et al. (2021) [27]	Tottori Prefecture, Japan	Residents Affected by Heat Strokes	1108	Case-Crossover Study	Logistic Regression, Ambulance Records, Meteorological Data	Incidence of Heat Strokes, Severity of Heat Strokes	Age, Air Temperature	Heat Stroke, Non-Exertional Heat Stroke	Summer Minimum/Maximum Temperatures	2017–2019 (Summer Months)
Fujitani et al. (2019) [28]	Tottori Prefecture, Japan	Residents Affected by Heat Strokes	382	Case-Crossover Study	Time-Stratified Case-Crossover Method, Meteorological Data	ATHS (Ambulance Transports for Heat Stroke), Effect of Maximum Air Temperature	Air Temperature (Hot Days vs. Cooler Days)	Heat Stroke Risk in Spring vs. Summer	Maximum Air Temperature, Seasonal Variations	April–September 2017
Ng et al. (2014) [25]	Kanto Region, Japan	Residents Affected by Heatstroke	12,907	Time-Series Study	Generalized Additive Models, Zero-Inflated Poisson Regression, Meta-Analysis	Heatstroke-Related Ambulance Dispatches (HSAD)	Air Temperature, Seasonal Variations	Heat Stroke Risk, Initial Warming Effects	Maximum Three-Hour Apparent Temperature (AT)	May–September (2000–2009)
Toosty et al. (2021) [29]	Fukuoka City, Japan	Residents Transported for Heatstroke		Epidemiological Study	Negative Binomial Regression, Age-Structure Analysis	Heatstroke Incidence, Risk Factors	Age (≥70 years), Wet-Bulb Globe Temperature, Mean & Time-Weighted Temperature	Heat Stroke (Severe Cases Requiring Emergency Transport)	Urban Heat Island, Extreme Heat Exposure	Summer (Year Not Specified)

**Table:** 

S/N	Author	Clear Statement of the research aims?	Appropriateness of qualitative methodology?	Appropriateness of research design?	Recruitment strategy appropriateness?	Data collection methods aligned with research question?	Researcher-participant relationship considered?	Ethical considerations addressed?	Rigorous data analysis?	Clear statement of findings?	Research value?	Total
1.	Adejoh et al. (2022)	Y	Y	Y	Y	Y	C	Y	Y	Y	Y	9/10
2.	Akanwa et al. (2022)	Y	Y	Y	Y	Y	Y	Y	Y	Y	Y	10/10
3.	Duru et al. (2022)	Y	Y	Y	C	Y	N	Y	Y	N	Y	7/10
4.	Micheal (2024)	Y	Y	Y	Y	Y	C	Y	Y	Y	Y	9/10

**Table:** 

S/N	Authors	Did the study address a clearly focused issue?	Was the cohort recruited in an acceptable way?	Was the exposure accurately measured to minimize bias?	Was the outcome accurately measured to minimize bias?	Have the authors identified all important confounding factors?	What are the results of this study?	How precise are the results?	How precise are the results?	Can the results be applied to the local population?	Do the results fit with other available evidence?	What are the implications for practice?	Total
1	Otani et al. (2021)	Y	Y	Y	Y	C	N	Y	Y	Y	Y	Y	10/12
2	Fujitani et al. (2019)	Y	Y	Y	Y	Y	Y	Y	Y	Y	Y	Y	12/12
3	Ng et al. (2014)	Y	C	Y	Y	N	N	Y	Y	Y	Y	Y	9/12
4	Toosty et al. (2021)	Y	Y	Y	Y	Y	Y	Y	Y	Y	Y	Y	12/12
